# *In Vitro* Activity of Delafloxacin and Finafloxacin against Mycoplasma hominis and *Ureaplasma* Species

**DOI:** 10.1128/spectrum.00099-22

**Published:** 2022-05-09

**Authors:** Yingying Kong, Chao Li, Gangfeng Li, Ting Yang, Mohamed S. Draz, Xinyou Xie, Jun Zhang, Zhi Ruan

**Affiliations:** a Department of Clinical Laboratory, Sir Run Run Shaw Hospital, Zhejiang University School of Medicine, Hangzhou, China; b Key Laboratory of Precision Medicine in Diagnosis and Monitoring Research of Zhejiang Province, Hangzhou, China; c Department of Medicine, Case Western Reserve University School of Medicine, Cleveland, Ohio, USA; d Department of Biomedical Engineering, Cleveland Clinic, Cleveland, Ohio, USA; University of Guelph

**Keywords:** *Mycoplasma hominis*, *Ureaplasma* spp., delafloxacin, finafloxacin, fluoroquinolone-resistant, in vitro activity

## Abstract

The *in vitro* activity of two new fluoroquinolones, delafloxacin and finafloxacin, were evaluated against *M. hominis* and *Ureaplasma* spp. The MICs of delafloxacin, finafloxacin, and two classical fluoroquinolones (moxifloxacin and levofloxacin) were tested against 29 *M. hominis* and 67 *Ureaplasma* spp. isolates using the broth microdilution method. The molecular mechanisms underlying fluoroquinolone resistance were also investigated. Delafloxacin exhibited low MICs against *M. hominis* and *Ureaplasma* spp., including the levofloxacin-resistant isolates. For *M. hominis*, delafloxacin showed low MIC_90_ value of 1 μg/mL (MIC range, <0.031 -1 μg/mL) compared to 8 μg/mL for finafloxacin, 16 μg/mL for moxifloxacin, and 32 μg/mL for levofloxacin. For *U. parvum* and *U. urealyticum*, delafloxacin had low MIC_90_ values (*U. parvum*, 2 μg/mL; *U. urealyticum*, 4 μg/mL) compared to 16 -32 μg/mL for finafloxacin, 16 μg/mL for moxifloxacin, and 32 - >32 μg/mL for levofloxacin. The two mutations GyrA S153L and ParC S91I were commonly identified in fluoroquinolone-resistant *M. hominis*, and ParC S83L was the most frequent mutation identified in fluoroquinolone-resistant *Ureaplasma* spp. Delafloxacin displayed lower MICs against fluoroquinolone-resistant isolates of both *M. hominis* and *Ureaplasma* spp. that have mutations in the quinolone resistance determining regions (QRDRs) than the two classical fluoroquinolones. Delafloxacin is a promising fluoroquinolone with low MICs against fluoroquinolone-resistant *M. hominis* and *Ureaplasma* spp. Our study confirms the potential clinical use of delafloxacin in treating antimicrobial-resistant *M. hominis* and *Ureaplasma* spp. infections.

**IMPORTANCE** Fluoroquinolone resistance in Mycoplasma hominis and *Ureaplasma* spp. is on the rise globally, which has compromised the efficacy of the currently available antimicrobial agents. This study evaluated the antimicrobial activity of two new fluoroquinolones, delafloxacin and finafloxacin, for the first time, against *M. hominis* and *Ureaplasma* spp. clinical isolates. Delafloxacin and finafloxacin displayed different antimicrobial susceptibility profiles against *M. hominis* and *Ureaplasma* spp. *in vitro*. Delafloxacin was found to be more effective against *M. hominis* and *Ureaplasma* spp. than three classical fluoroquinolones (finafloxacin, moxifloxacin, and levofloxacin). Finafloxacin displayed activity similar to moxifloxacin but superior to levofloxacin against *M. hominis* and *Ureaplasma* spp. Our findings demonstrate that delafloxacin is a promising fluoroquinolone with outstanding activity against fluoroquinolone-resistant *M. hominis* and *Ureaplasma* spp.

## INTRODUCTION

Mycoplasma hominis and *Ureaplasma* species (*U. parvum* and *U. urealyticum*) are members of the class of *Mollicutes* and common commensals colonizing in the urogenital tract of humans. However, they have been associated with chorioamnionitis, adverse pregnancy outcomes, infertility, and neonatal diseases in some instances (1–3). Both *M. hominis* and *Ureaplasma* spp. lack cell wall; thus, antimicrobial treatments are limited to those that can inhibit DNA replication (e.g., fluoroquinolones) and protein synthesis (e.g., macrolides and tetracyclines). An increasing number of reports confirmed the presence of antimicrobial resistance in clinical *M. hominis* and *Ureaplasma* spp. isolates, especially against fluoroquinolones. Recent surveillance studies reported that fluoroquinolone resistance in *M. hominis* and *Ureaplasma* spp. isolates recovered from Chinese patients is on the rise, which has compromised the efficacy of the currently available fluoroquinolones (4–6). In our previous study, > 80% *M. hominis* and > 50% *Ureaplasma* spp. isolates were resistant to levofloxacin and moxifloxacin, respectively (4). Therefore, new fluoroquinolones against *M. hominis* and *Ureaplasma* spp. are urgently needed to establish effective treatment.

In *M. hominis* and *Ureaplasma* spp., amino acid mutations in quinolone resistance determining regions (QRDRs), including DNA gyrase (GyrA and GyrB) and the topoisomerase IV complex (ParC and ParE), are a key mechanism of fluoroquinolone resistance (7). Delafloxacin and finafloxacin are novel fluoroquinolones with improved antibacterial activity against various infections. Delafloxacin is a new dual-targeting anionic fluoroquinolone with modifications at carbon C-7 and C-8 (8), and its unique molecular structure increases its intracellular penetration and potency against multidrug-resistant bacteria - especially under acidic environments (9–11). Unlike other fluoroquinolones, delafloxacin can target DNA gyrase and topoisomerase IV equally, decreasing the selection of resistant mutations. Finafloxacin is another novel fluoroquinolone with 8-cyano and C-7 substituents, which also inhibits topoisomerase IV and DNA gyrase (12, 13). Therefore, both delafloxacin and finafloxacin can be effectively used in treating infections caused by a broad spectrum of organisms, including those with mutations in the QRDRs.

In this study, we evaluated the activity of delafloxacin and finafloxacin *in vitro* against *M. hominis* and *Ureaplasma* spp., compared with two classical fluoroquinolones (moxifloxacin and levofloxacin) using the broth microdilution method. We also identified the mutations in QRDRs and evaluated their associations with the MICs of delafloxacin and finafloxacin.

## RESULTS

### MICs of the four fluoroquinolones against *M. hominis* and *Ureaplasma* spp. isolates.

The MIC distribution, MIC_50,_ and MIC_90_ values, and the rates of antimicrobial resistance for the four fluoroquinolones (delafloxacin, finafloxacin, moxifloxacin, and levofloxacin) are determined and represented in Table 1.

**TABLE 1:** MIC distributions of delafloxacin, finafloxacin, moxifloxacin, and levofloxacin against *M. hominis* and *Ureaplasma* species^*a*^

Organism and antimicrobials	No. of isolates with the indicated MIC (μg/mL)														MIC_50_ (μg/mL)	MIC_90_ (μg/mL)	Resistance %
	**<0.031**	**0.031**	**0.063**	**<0.125**	**0.125**	**0.25**	**0.5**	**1**	**2**	**4**	**8**	**16**	**32**	**>32**			
*M. hominis* (*n* = 29)																	
Delafloxacin	6		1			3	12	7							0.5	1	NA
Finafloxacin	5						1	1		4	16	2			8	8	NA
Moxifloxacin				5				1	3	1	3	15	1		16	16	24 (82.76%)
Levofloxacin							2		1	2	3	3	17	1	32	32	27 (93.10%)
*U. parvum* (*n* = 52)																	
Delafloxacin	5		1		10	2	4	21	6	3					1	2	NA
Finafloxacin							1	7	7	6	20	10	1		8	16	NA
Moxifloxacin							3	6	6	19	8	9	1		4	16	37 (71.15%)
Levofloxacin								5	6	7	11	12	9	2	8	32	41 (78.85%)
*U. urealyticum* (*n* = 15)																	
Delafloxacin					1	1		1	7	5					2	4	NA
Finafloxacin									2		1	10	2		16	32	NA
Moxifloxacin								2		1	4	7	1		16	16	13 (86.67%)
Levofloxacin									2			4	6	3	32	>32	13 (86.67%)

a For *M. hominis*, the breakpoints were ≥ 2 μg/mL and ≥ 0.5 μg/mL for levofloxacin and moxifloxacin, respectively. For *Ureaplasma* spp., the breakpoints were ≥ 4 μg/mL for levofloxacin and moxifloxacin. NA, not applicable (no CLSI breakpoint).

For the 29 *M. hominis* isolates tested, delafloxacin showed low MICs (MIC_50_ = 0.5 μg/mL; MIC_90_ = 1 μg/mL) compared to finafloxacin, moxifloxacin, and levofloxacin, with MICs ranging from < 0.031 μg/mL to 1 μg/mL. However, finafloxacin, another new fluoroquinolone, showed relatively high MIC_50_ and MIC_90_ values (MIC_50_ = 8 μg/mL; MIC_90_ = 8 μg/mL), with MICs ranging from < 0.031 μg/mL to 16 μg/mL. According to the CLSI guideline, 82.76% (24/29) and 93.1% (27/29) isolates exhibited resistance to moxifloxacin and levofloxacin, respectively, with MIC_90_ values of 16 μg/mL for moxifloxacin and of 32 μg/mL for levofloxacin.

Of the 67 *Ureaplasma* spp. tested, delafloxacin also exhibited low MIC_50_ (*U. parvum*, 1 μg/mL; *U. urealyticum*, 2 μg/mL) and MIC_90_ (*U. parvum*, 2 μg/mL; *U. urealyticum*, 4 μg/mL) values, with MICs ranging from < 0.031 μg/mL to 4 μg/mL for *U. parvum* and from 0.125 μg/mL to 4 μg/mL for *U. urealyticum*. In contrast, finafloxacin exhibited high values of MIC_50_ (*U. parvum*, 8 μg/mL; *U. urealyticum*, 16 μg/mL) and MIC_90_ (*U. parvum*, 16 μg/mL; *U. urealyticum*, 32 μg/mL) in both *U. parvum* and *U. urealyticum*. The resistance rate of moxifloxacin ranged from 71.15% (37/52) in *U. parvum* to 86.67% (13/15) in *U. urealyticum*, with elevated MIC_50_ values (*U. parvum*, 4 μg/mL; *U. urealyticum*, 16 μg/mL) and the same MIC_90_ values (16 μg/mL). Levofloxacin resistance was found in 78.85% (41/52) *U. parvum* and 86.67% (13/15) *U. urealyticum* isolates, with elevated MIC_50_ (*U. parvum*, 8 μg/mL; *U. urealyticum*, 32 μg/mL) and MIC_90_ values (*U. parvum*, 32 μg/mL; *U. urealyticum*, > 32 μg/mL).

### Molecular mechanism of fluoroquinolone resistance.

The nucleotide and deduced amino acid sequences of the QRDRs of *gyrA*, *gyrB*, *parC*, and *parE* for all the 29 clinical *M. hominis* and 67 clinical *Ureaplasma* spp. isolates were compared with reference strains of *M. hominis* and *Ureaplasma* spp., respectively. In general, levofloxacin-resistant strains demonstrated reduced susceptibilities to delafloxacin, finafloxacin, and moxifloxacin compared to those of levofloxacin-susceptible strains of the same species (Tables 2 and 3).

**TABLE 2:** The MICs and genetic alterations of *M. hominis*

	Delafloxacin MIC (μg/mL)		Finafloxacin MIC (μg/mL)		Moxifloxacin MIC (μg/mL)		Levofloxacin MIC (μg/mL)		Genetic alteration^*b*^			
No. of isolates^*a*^	Range	MIC_50_	Range	MIC_50_	Range	MIC_50_	Range	MIC_50_	GyrA	GyrB	ParC	ParE
*M. hominis* (*S* = 2)												
2	<0.031		<0.063		<0.125		0.5		N	N	N	N
*M. hominis* (*R* = 27)												
1	<0.031		<0.063		<0.125		2		N	N	N	N
2	<0.031-1		<0.063-8		<0.125-8		4-16				G272T(S91I)	*G1249A(V417I)*
1	0.5		4		2		8				*A431G(K144R)*	
1	1		8		2		8				*A431G(K144R)*	*G1249A(V417I)*
1	<0.031		<0.063		<0.125		8					*G1249A(V417I)* & G1276A(D426N)
1	0.0625		1		2		4		C458T(S153L）		**T274C(S92P)** & *A431G(K144R)*	
2	<0.031-0.5		0.5-4		1-4		16−32		T457G(S153A)		G272T(S91I)	*G1249A(V417I)*
1	0.5		8		16		32		C458T(S153L)		G272T(S91I)	
1	0.5		8		16		32		C458T(S153L)		G272T(S91I) & *A431G(K144R)*	
4	0.5-1	0.5	8-16	8	16-32	16	32	32	C458T(S153L)		G272T(S91I)	*G1249A(V417I)*
10	0.25-1	0.5	4-8	8	8-16	16	32->32	32	C458T(S153L)		G272T(S91I) & *A431G(K144R)*	*G1249A(V417I)*
1	1		8		8		16		C458T(S153L)		G272T(S91I) & *A431G(K144R)* & **G460A(A154T)**	*G1249A(V417I)*
1	0.5		4		16		32		C458T(S153L)	C1418T(A473V)	G272T(S91I)	
ATCC 23114	<0.031		0.5		0.25		0.5					

a *S*, susceptible; *R*, resistant.

b N, no mutation detected. Numbers and letters in parentheses indicate amino acid substitutions that occurred because of DNA point mutations. Novel mutations are highlighted in bold. Mutations unrelated to fluoroquinolone resistance are identified in italic.

**TABLE 3:** The MICs and genetic alterations of *U. parvum* and *U. urealyticum*

	Delafloxacin MIC (μg/mL)		Finafloxacin MIC (μg/mL)		Moxifloxacin MIC (μg/mL)		Levofloxacin MIC (μg/mL)		Genetic alteration^*b*^			
No. of isolates^*a*^	Range	MIC_50_	Range	MIC_50_	Range	MIC_50_	Range	MIC_50_	GyrA	GyrB	ParC	ParE
*U. parvum* (*S* = 11)												
4	0.125	0.125	1-2	1	0.5-1	0.5	1-2	1	N	N	N	N
1	0.125		1		0.5	1					*T250C(S84P)*	
1	<0.031		0.5		1		1				*G310A(D104N)*	
1	0.25		2		2		2				*T331A(S111T)*	
4	<0.031-0.125	0.125	1	1	1-2	1	1-2	2			*G406T(A136S)*	
*U. parvum* (*R* = 41)												
2	1		8		4-16		8-32		N	N	N	N
5	<0.031-0.125	0.063	2-4	2	2-4	4	4	4				G1343A(R448K)
1	<0.031		2		2		4				*G406T(A136S)*	G1343A(R448K)
2	0.25-1		4-16		4-16		8-32				G259A(E87K)	
1	0.5		4		8		8				T247G(S83A)	
24	0.5-2	1	4-16	8	2-16	4	4-32	16			C248T(S83L)	
1	2		8		8		16			A1442G(N481S)	C248T(S83L)	
1	0.5		8		8		16			C1384T(P462S)	C248T(S83L)	
1	0.5		16		16		32			A1446C(E482D)	C248T(S83L)	
1	4		16		16		32				C248T(S83L)	G1441A(A481T)
1	4		4		16		>32			A1328C(D443A)	C248T(S83L)	
1	4		32		32		>32		C310A(Q104K)		C248T(S83L)	
*U. urealyticum* (*S* = 2)												
2	0.125-0.25		2		1		2		N	N	N	N
*U. urealyticum* (*R* = 13)												
1	2		16		8		16		N	N	N	N
	1-4	2	16-32	16	4-16	16	16->32	32			C248T(S83L)	
1	2		8		16		32			A1456T(N486Y)	C248T(S83L)	
1	4		32		32		>32		A299T(Q100L)		C248T(S83L)	
ATCC 27618	<0.031		0.5		0.5		2					

a *S*, susceptible; *R*, resistant.

b N, no mutation detected. Numbers and letters in parentheses indicate amino acid substitutions that occurred because of DNA point mutations. Mutations unrelated to fluoroquinolone resistance are identified in italic.

Of the 29 *M. hominis*, the mutations of K144R in ParC and V417I in ParE, unrelated to fluoroquinolone resistance, were observed in 24 resistant isolates. Significant differences in MICs of fluoroquinolones were observed in the two isolates that harbored the ParC S91I mutation and the two isolates that harbored double mutations GyrA S153A/ParC S91I. Sixteen isolates, harbored the double mutations GyrA S153L/ParC S91I, displayed increased MIC_50_ values for delafloxacin (0.5 μg/mL), finafloxacin (8 μg/mL), moxifloxacin (16 μg/mL), and levofloxacin (32 μg/mL). Both the isolate harbored GyrA S153L/ParC S91I/ParC A154T mutations and the isolate harbored GyrA S153L/GyrB A473V/ParC S91I mutations displayed similar MICs with the above 16 isolates.

Among the 11 levofloxacin-sensitive *U. parvum* isolates, 7 isolates had a single mutation in ParC, including D104N, S111T, or A136S. For the five isolates that harbored a single mutation of R448K in ParE, the delafloxacin exhibited a low MIC_50_ value of 0.063 μg/mL compared to 2 μg/mL for finafloxacin, 4 μg/mL for moxifloxacin, and 4 μg/mL for levofloxacin. One isolate harboring the double mutations ParC A136S/ParE R448K showed similar MICs to the isolates harboring ParE R448K. For the 24 isolates harboring a single mutation of S83L in ParC, the delafloxacin also showed a low MIC_50_ value of 1 μg/mL compared to finafloxacin (8 μg/mL), moxifloxacin (4 μg/mL), and levofloxacin (16 μg/mL). For the six isolates harboring double mutations of ParC S83L along with GyrA Q104K, GyrB (N481S, P462S, E482D, or D443A), or ParE A481T, the MIC_50_ values of the four fluoroquinolones (delafloxacin, 2 μg/mL; finafloxacin, 8 μg/mL; moxifloxacin, 16 μg/mL; levofloxacin, 32 μg/mL) were comparable to the isolates with a single mutation of ParC S83L.

Of the 13 levofloxacin-resistant *U. urealyticum*, mutations in the QRDRs were identified in 12 isolates. 10 isolates had a single mutation of S83L in ParC. Compared to *U. parvum* with the same mutation, the MIC_50_ values of the four fluoroquinolones (delafloxacin, 2 μg/mL; finafloxacin, 16 μg/mL; moxifloxacin, 16 μg/mL; levofloxacin, 32 μg/mL) against *U. urealyticum* increased 2- to 4-fold. Two isolates carrying ParC S83L and GyrA Q100L or GyrB N486Y showed similar MICs to the ParC S83L harboring isolates.

## DISCUSSION

Increasing fluoroquinolones resistance in *M. hominis* and *Ureaplasma* spp. has been well-documented and dramatically limits the clinical treatment options. This study compared the *in vitro* activity of two new fluoroquinolones, delafloxacin and finafloxacin, against *M. hominis* and *Ureaplasma* spp.

In the present study, high levels of moxifloxacin and levofloxacin resistance against *M. hominis* and *Ureaplasma* spp. were identified, in which 93.1% *M. hominis*, 78.85% *U. parvum*, and 86.67% *U. urealyticum* showed levofloxacin-resistant, and 82.76% *M. hominis*, 71.15% *U. parvum*, and 86.67% *U. urealyticum* were moxifloxacin-resistant. A high level of fluoroquinolone resistance was discovered in *M. hominis* and *Ureaplasma* spp. in different parts of China. Zhang et al. showed that 85.7% of *M. hominis* isolates were levofloxacin-resistant, and 73.8% were moxifloxacin-resistant (14). Ma et al. showed that 72.87% of *Ureaplasma* spp. were levofloxacin-resistant, and 89.92% were ciprofloxacin-resistant (5). In our previous study, the resistance rate of moxifloxacin was 25.3% for *U. parvum* and 50% for *U. urealyticum*, and that of levofloxacin was 75.9% for *U. parvum* and 71.4% for *U. urealyticum*, suggesting an increasing trend of moxifloxacin and levofloxacin resistance against *Ureaplasma* spp. in recent years (15). It was noteworthy that fluoroquinolones resistance levels varied significantly between countries. In France, the levofloxacin and moxifloxacin resistance rates of *Ureaplasma* spp. were 1.2% and 0.1%, respectively, and those for *M. hominis* were 2.7% and 1.6%, respectively (16). In the United States, low levofloxacin-resistant rates (1.6% for *U. parvum*, 0% for *U. urealyticum*, and 0% for *M. hominis*) were observed (17). In contrast, a high fluoroquinolone resistance has been identified in Japan (57.15% levofloxacin resistance rate for *Ureaplasma* spp.) (18). Fluoroquinolone resistance in *M. hominis* and *Ureaplasma* spp. has been extremely high in China, perhaps due to the inappropriate fluoroquinolone agents in both clinical settings and the poultry industry (19, 20).

Delafloxacin and finafloxacin are novel synthetic anionic fluoroquinolones with quinolone structure modifications to improve antibacterial effectiveness, pharmacokinetic profile, and toxicity profile and have been approved by the US Food and Drug Administration (FDA). Both delafloxacin and finafloxacin exhibited a broad spectrum of activity against Gram-positive and Gram-negative bacteria (8–13). However, to our knowledge, until now, no studies have been undertaken in China to determine the *in vitro* activity of delafloxacin and finafloxacin against *M. hominis* and *Ureaplasma* spp. using the current CLSI guidelines. In this study, delafloxacin and finafloxacin displayed different antimicrobial susceptibility profiles against *M. hominis* and *Ureaplasma* spp. *in vitro*. Delafloxacin was found to be more effective against *M. hominis* and *Ureaplasma* spp. than the other three fluoroquinolones (finafloxacin, moxifloxacin, and levofloxacin). Finafloxacin displayed activity similar to moxifloxacin but superior to levofloxacin against *M. hominis* and *Ureaplasma* spp.

The study by Waites and colleagues tested the antimicrobial activity of multiple fluoroquinolones, including delafloxacin, finafloxacin, moxifloxacin, and levofloxacin, and showed low MIC_90_ values against 10 clinical *M. hominis* isolates (delafloxacin, 0.016 μg/mL; finafloxacin, 0.063 μg/mL; moxifloxacin, 0.5 μg/mL; levofloxacin, 0.5 μg/mL) and 22 clinical *Ureaplasma* spp. isolates (delafloxacin, 0.25 μg/mL; finafloxacin, 0.5 μg/mL; moxifloxacin, 2 μg/mL; levofloxacin, 2 μg/mL) collected from the US in 2003 (21). However, relatively higher MIC_90_ values of fluoroquinolones against *M. hominis* (delafloxacin, 1 μg/mL; finafloxacin, 8 μg/mL; moxifloxacin, 16 μg/mL; levofloxacin, 32 μg/mL) and *Ureaplasma* spp. (delafloxacin, 2 - 4 μg/mL; finafloxacin, 16 - 32 μg/mL; moxifloxacin, 16 μg/mL; levofloxacin, 32 - >32 μg/mL) were observed in this study. Considering the high resistance levels to moxifloxacin and levofloxacin against *M. hominis* and *Ureaplasma* spp. in China, delafloxacin may provide an alternative for developing an effective regimen against *M. hominis* and *Ureaplasma* spp. infections.

Resistance to fluoroquinolones is associated with genetic mutations in the QRDRs in *M. hominis* and *Ureaplasma* spp., including GyrA/B and ParC/E, which serve as the target of most fluoroquinolones. Several investigations have identified the mutations in the ofloxacin/ciprofloxacin/levofloxacin/moxifloxacin-resistant mutants, such as ParC S91I and GyrA S153L in *M. hominis*, and ParC S83L and ParE R448K in *Ureaplasma* spp. (4, 14, 15, 18, 22, 23). For *M. hominis*, mutations were identified in QRDRs of the 26 out of 27 levofloxacin-resistant isolates. These mutations contained S153L and S153A in GyrA; A473V in GyrB; S91I, K144R, S92P, and A154T in ParC; and V417I and D426N in ParE. However, a previous study identified ParC K144R and ParE V417I mutations unrelated to fluoroquinolone resistance (4). For *Ureaplasma* spp., 51 out of 54 levofloxacin-resistant isolates harbored amino acid changes in QRDRs, including Q100L and Q104K in GyrA; N486Y, N481S, P462S, E482D, and D443A in GyrB; E87K, S83A, and S83L in ParC; and R448K and A481T in ParE. Notably, S84P, D104N, S111T, and A136S mutations in ParC were observed in fluoroquinolone susceptible *Ureaplasma* spp. isolates, suggesting that these mutations were not involved in fluoroquinolone resistance. The MICs of delafloxacin were lower than finafloxacin and the two classical fluoroquinolones against levofloxacin-resistant isolates of *M. hominis* and *Ureaplasma* spp., although all the four fluoroquinolones demonstrated reduced activities in comparison to those of levofloxacin-susceptible isolates of the same species. Although delafloxacin may be affected by mutations in QRDRs, the *in vitro* antibacterial activity of delafloxacin is still significantly higher than other fluoroquinolones, including finafloxacin, moxifloxacin, and levofloxacin.

In conclusion, this study demonstrates the spectrum of activity of delafloxacin and finafloxacin against fluoroquinolone-resistant *M. hominis* and *Ureaplasma* spp. This study suggests that delafloxacin could be a promising therapeutic option for the treatment of *M. hominis* and *Ureaplasma* spp. infections, including those caused by fluoroquinolone-resistant isolates. More comprehensive and large-scale surveillance studies are warranted to better understand the antimicrobial susceptibility patterns of new fluoroquinolones.

## MATERIALS AND METHODS

### Bacterial strains and clinical specimens.

During the period of September 2018 to July 2019, 67 *Ureaplasma* spp. (52 Ureaplasma parvum and 15 Ureaplasma urealyticum) and 29 *M. hominis* isolates were cultured from clinical patients at Sir Run Run Shaw Hospital, Zhejiang University School of Medicine, China. Among these isolates, 70 isolates were recovered from the urogenital tracts of females aged 21–63 years, and 26 isolates were collected from the urogenital tracts of males aged 22–44 years.

### Antimicrobial agents and antimicrobial susceptibility testing.

Delafloxacin was obtained from WuXi AppTec (Wuhan, China). Finafloxacin, moxifloxacin, and levofloxacin were purchased from Sigma-Aldrich (St. Louis, MO, USA). Antimicrobial agents were dissolved according to the manufacturers’ instructions. Delafloxacin was dissolved in dimethyl sulfoxide (DMSO), and the other three fluoroquinolones were dissolved in deionized water. The MICs of four fluoroquinolones (delafloxacin, finafloxacin, moxifloxacin, and levofloxacin) were determined in duplicate by the microdilution broth method according to the guidelines of the CLSI. The concentration ranges for moxifloxacin and levofloxacin was from 0.125 μg/mL to 32 μg/mL, while those for finafloxacin and delafloxacin were from 0.063 μg/mL to 32 μg/mL and from 0.031 μg/mL to 16 μg/mL, respectively. The breakpoints were interpreted according to CLSI guideline M43-A. *M. hominis* ATCC 23114 and *U. urealyticum* ATCC 27618 were used as reference strains.

### Identification of *Ureaplasma* species and genetic mechanisms of fluoroquinolone resistance.

Zero-point five milliliters (0.5 mL) of a broth culture of *M. hominis* or *Ureaplasma* spp. strain was used to isolate genomic DNA. To distinguish *U. parvum* from *U. urealyticum*, amplification was conducted with *Ureaplasma* species, as previously described (15). Amplifications of the QRDRs of *M. hominis* and *Ureaplasma* spp. isolates were conducted using previously reported primers (22–24). Purified PCR products were sequenced and then mapped to the corresponding sequences of *U. parvum* ATCC 700970, *U. urealyticum* ATCC 33699, or *M. hominis* ATCC 23114.
